# Nail involvement due to immunoglobulin G4 (IgG4)-related disease in a person living with HIV^⋆^^☆^

**DOI:** 10.1016/j.abd.2024.09.007

**Published:** 2025-04-07

**Authors:** Valéria Lukenczuk Said, Monique Freire dos Reis, Tiago Vencato da Silva, Virgínia Vilasboas Figueiras, Nathália Matos Gomes

**Affiliations:** a Department of Dermatology, Fundação de Medicina Tropical Doutor Heitor Vieira Dourado, Manaus, AM, Brazil; b Department of Pathology and Forensic Medicine, Universidade Federal do Amazonas, Manaus, AM, Brazil; c Laboratório de Patologia Bacchi Ltda, Botucatu, SP, Brazil

Dear Editor,

Immunoglobulin-4 (IgG4)-related disease is an immune-mediated disorder characterized by the presence of a lymphoplasmacytic infiltrate rich in IgG4-positive tissue plasma cells and elevated serum IgG4. This condition causes inflammation with fibrosis and presents with symptoms that vary depending on the affected organ. It usually affects the pancreas, salivary glands, lacrimal glands, biliary tract, and peritoneum. Cutaneous involvement is rare and occurs most frequently in the jaw, mental protuberance, and cervical region, and this is the first description in the literature involving the nail apparatus.^1^

A 47-year-old man, living with HIV, with a CD4 count of 349 cells and an undetectable viral load, complained of progressive nail changes over the past two months, which were asymptomatic and did not improve after self-medication with ointments. In his past history, he had been previously treated for pulmonary tuberculosis and onychomycosis. On examination, edema was observed in the proximal nail fold, with ulceration of the nail bed and complete absence of the nail plate of the fourth finger of the right hand, in addition to onychodystrophy in the other nails (Fig. 1). Onychoscopy of the lesion showed irregular vascularization of the nail bed (Fig. 2). There were no other skin lesions or alterations in the lymphatic, cardiovascular, respiratory or gastrointestinal tract systems.

**Fig. 1:**
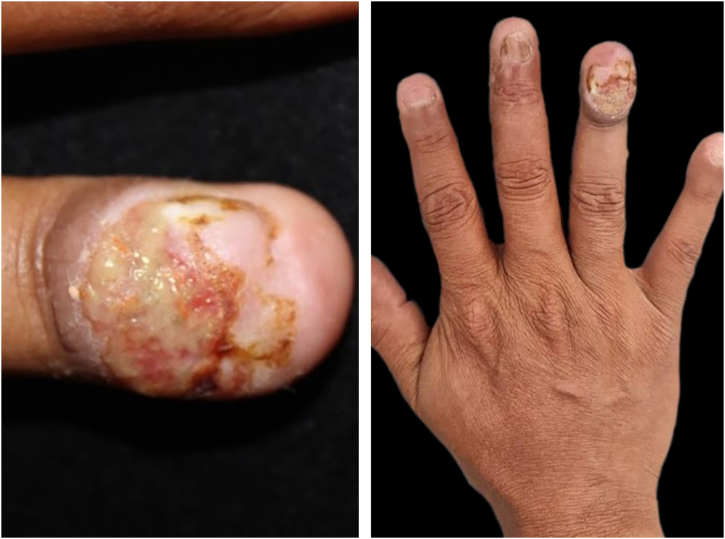
Clinical manifestation of IgG4-related disease in the nail: (A) significant involvement of the nail apparatus with edema in the proximal nail fold, nail bed ulceration, and complete absence of the nail plate. (B) Onychodystrophy is seen in the other nail plates.

**Fig. 2:**
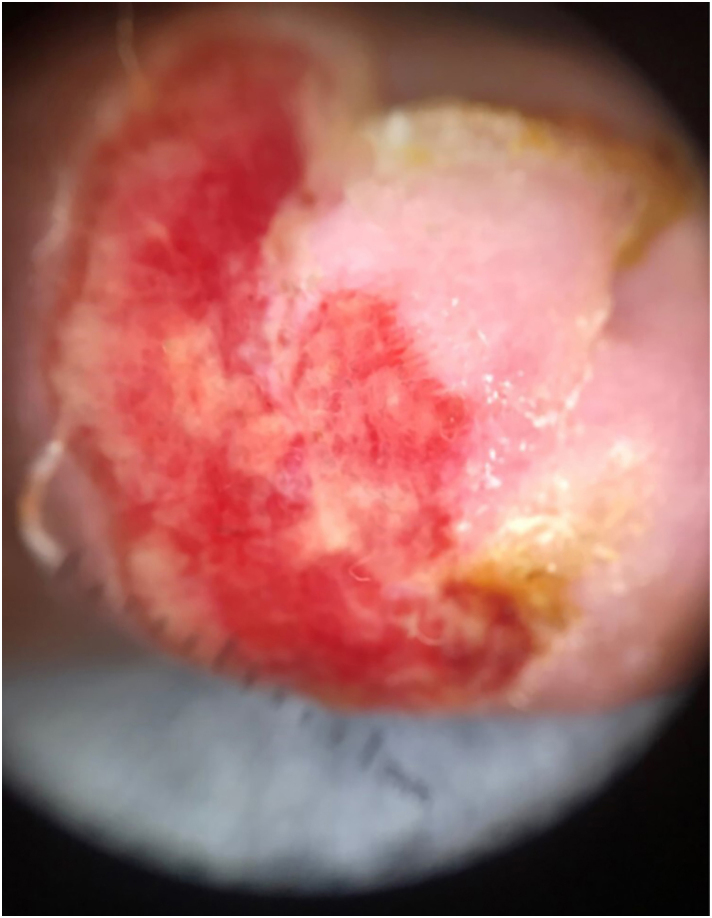
Onychoscopy of IgG4-related nail disease: erosion and irregular vascularization of the nail bed.

A skin biopsy was performed and sent for tissue microscopy, culture and histopathology. The microbiological examination was negative for fungi and AFB. The histopathological examination showed ulceration and a lymphoplasmacytic infiltrate in the connective tissue, interspersed with granulocytes, eosinophils and extensive areas of fibrosis. Immunohistochemistry confirmed the presence of IgG4+ plasma cells > 200/high power field (HFP), with an IgG4+/IgG ratio > 40% (Fig. 3). Additional histochemistry did not reveal fungal or bacterial organisms. Moreover, serum IgG4 was increased: 434 mg/dL. Laboratory tests, with the exception of ESR (10 mm), were normal or negative: complete blood count; biochemistry; VDRL; Anti-LA; C-anca; P-anca; Anti-DNA and CRP. There was no bone involvement on the X-ray. Computed tomography scans of the chest, abdomen and pelvis were performed, which detected architectural distortion in the lung apices, due to bands of atelectasis and bronchiectasis in between, in addition to multiple calcified retroperitoneal lymph node enlargements, suggesting previous granulomatous infection. The patient underwent two sessions of intralesional infiltration with triamcinolone acetonide 2.5 mg/mL, after which there was complete improvement of the lesion, without loss of limb functionality (Fig. 4) and continues to be monitored by a multidisciplinary team.

**Fig. 3:**
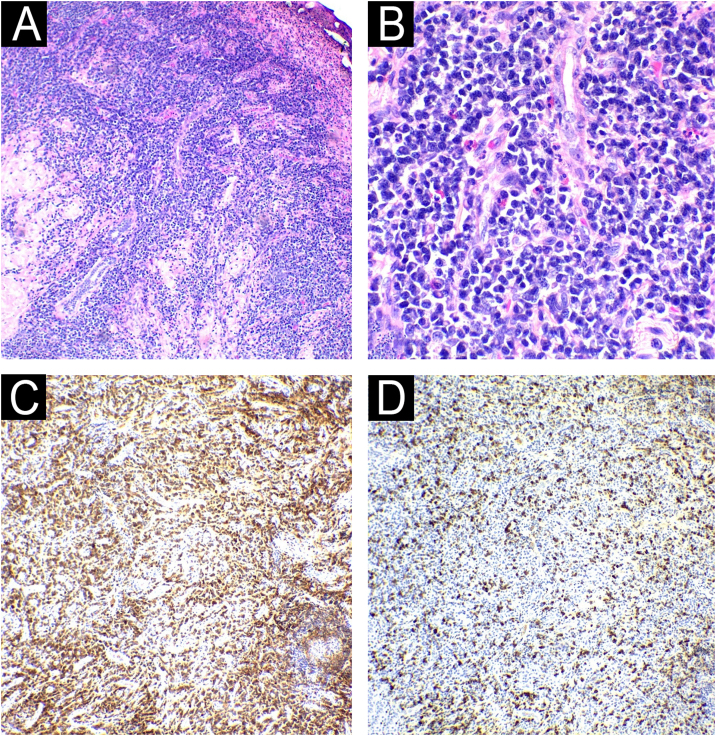
Histopathology of the nail lesion: (A) Ulceration and dense perivascular, periadnexal and interstitial inflammatory infiltrate in the connective tissue, both superficial and deep, amid areas of fibrosis (Hematoxylin & eosin ×40). (B) Inflammatory infiltrate consisting predominantly of plasma cells, interspersed with lymphocytes and eosinophils. (Hematoxylin & eosin ×100). Immunohistochemistry showing an increased IgG4/IgG ratio with >200 IgG4-positive plasma cells per high-power field (in C, IgG; in D, IgG4).

**Fig. 4:**
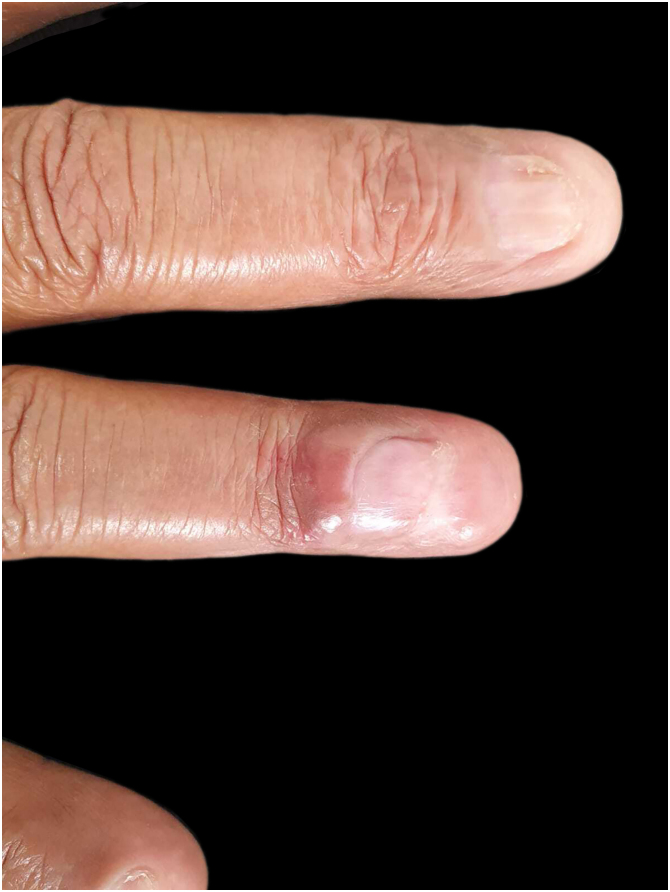
Result of treatment of IgG4-related nail disease: significant improvement with functional maintenance of the finger.

Cutaneous involvement in IgG4-related disease refers to tumor lesions due to a local inflammatory process in the skin. The prevalence of cutaneous lesions in this condition varies from 4.2% to 6.3% and they are most commonly described as subcutaneous papules, plaques or nodules in the head and neck region associated with systemic disease, in middle-aged men.^2^ Primary presentation in the skin, as in the present case, is extremely rare.^3^ Recently, a similar case was reported in the literature of a patient living with HIV, with the cutaneous form of IgG4-related disease, showing a single ulcerated lesion in the inguinal region, without any involvement of other organs.^4^

According to the 2020 Consensus Review and Diagnostic Criteria for IgG4-related disease, the following criteria must be present to establish its diagnosis: 1) Clinical and radiological characteristics: one or more organs demonstrating diffuse or localized edema or a characteristic mass or nodule; 2) Serological diagnosis: serological IgG4 levels > 135 mg/dL; and 3) Pathological diagnosis: two of the following findings: a) Dense infiltrate of lymphocytes and plasma cells with fibrosis; b) IgG4+/IgG plasma cell ratio > 40% and number of IgG4 cells > 10 per high-power field; c) Presence of storiform fibrosis or obliterative phlebitis.^2^ The diagnosis is definitive when it includes all three domains; probable, when it includes only the clinical and pathological criteria; and possible, when it includes the clinical and serological criteria.^5^ Since the patient met all the criteria a definitive diagnosis of IgG4-related disease was established.

Systemic steroids are the first-line agents for inducing disease remission, but they present high recurrence rates after discontinuation. Other treatments previously described include surgical excision, topical corticosteroids, azathioprine, rituximab, and methotrexate.^6^, ^7^, ^8^ Since the reported patient showed only nail involvement, it was believed that local therapy would be sufficient, and systemic therapy was not necessary. The patient continues to be monitored by a multidisciplinary team.

In summary, by describing this atypical case, the authors expect to contribute to increasing the diagnostic suspicion of IgG4-related disease regarding the differential diagnoses of nail diseases. Additionally, the importance of accurate clinical-pathological correlation to attain a precise diagnosis is reinforced.

## Financial support

None declared.

## Authors’ contributions

Valéria Lukenczuk Said: Design and planning of the study; drafting and editing of the manuscript; collection, analysis and interpretation of data; statistical analysis; intellectual participation in the propaedeutic and/or therapeutic conduct of the studied cases; critical review of the literature.

Monique Freire: Critical review of the literature; collection, analysis and interpretation of data; effective participation in research orientation; approval of the final version of the manuscript.

Tiago Vencato da Silva: Analysis and interpretation of data; approval of the final version of the manuscript.

Virginia Vilasboas Figueiras: Critical review of the literature; analysis and interpretation of data; approval of the final version of the manuscript.

Nathalia Matos Gomes: Effective participation in research orientation; intellectual participation in the propaedeutic and/or therapeutic conduct of the studied cases; critical review of the literature; approval of the final version of the manuscript.

## Conflicts of interest

None declared.
